# Novel *Treponema pallidum* Recombinant Antigens for Syphilis Diagnostics: Current Status and Future Prospects

**DOI:** 10.1155/2017/1436080

**Published:** 2017-04-24

**Authors:** Aleksey Kubanov, Anastassia Runina, Dmitry Deryabin

**Affiliations:** Department of Laboratory Diagnostics of Sexually Transmitted Diseases and Dermatoses, State Research Center of Dermatovenereology and Cosmetology, Korolenko Street 3/6, Moscow 107076, Russia

## Abstract

The recombinant protein technology considerably promoted the development of rapid and accurate treponema-specific laboratory diagnostics of syphilis infection. For the last ten years, the immunodominant recombinant inner membrane lipoproteins are proved to be sensitive and specific antigens for syphilis screening. However, the development of an enlarged* T. pallidum *antigen panel for diagnostics of early and late syphilis and differentiation of syphilis stages or cured syphilis remains as actual goal of multidisciplinary expertise. Current review revealed novel recombinant antigens: surface-exposed proteins, adhesins, and periplasmic and flagellar proteins, which are promising candidates for the improved syphilis serological diagnostics. The opportunities and limitations of diagnostic usage of these antigens are discussed and the criteria for selection of optimal antigens panel summarized.

## 1. *Treponema pallidum* Biology

*Treponema pallidum* belongs to the family* Spirochaetaceae*, order* Spirochaetales*, phylum* Spirochaetes*, which is a phylogenetically ancient and distinct group of bacteria. Due to the cell structure, physiology, genetics, and pathogenic features* T. pallidum* is a very unusual microorganism [1].

*T. pallidum *is a Gram-negative spiral-shaped bacterium, which varies in length from 5 to 15 *μ*m and is 0,20 *μ*m in diameter.* T. pallidum* is covered with the outer membrane (OM), periplasmic space with endoflagella, peptidoglycan layer, and inner membrane (IM), which surrounds a cytoplasmic cylinder [2]. Three to six flagella extend in periplasmic space from both ends toward the centre of microorganism and determine the helical shape and characteristic corkscrew motility (rotating around longitudinal axis) of* T. pallidum *cells. This motility allows* T. pallidum* to permeate through membranous and gel-like substances and is important for* T. pallidum* invasion and dissemination during the syphilitic infection.

*T. pallidum* is a microaerophilic bacterium with an optimal growth temperature of 37°C and minimal metabolic capabilities. The microorganism is able to carry out glycolysis and interconversion of amino acids and fatty acids but lacks tricarboxylic acid cycle and alternative carbon sources pathways; de novo synthesis of amino acid, fatty acid, and nucleotides or enzyme cofactors synthesis pathways are also absent [3]. As a result,* T. pallidum* utilizes most of the essential molecules and substrates from the host environment using numerous specific transporters and does not survive outside the mammalian host [4].* T. pallidum* is a strictly extracellular pathogen, which is in a direct contact with the humoral and cellular immunity mechanisms and therefore can evade elimination and migrate into the immunologically privileged tissues of the host organism.

In fact, fastidious nature of this bacterium is a probable result of its long-term evolution and adaptation to the host environment [5], and that made* T. pallidum* one of the most dangerous human pathogens since 1495 till the development of antibiotic therapy.

## 2. *Treponema pallidum* Genome

A reference strain of* T. pallidum *subsp*. pallidum *is* Nichols*, which was isolated in 1912 from the cerebrospinal fluid of syphilitic patient in Washington DC and then was cultivated in rabbit testes. The novel reference strain, which is phenotypically distinct from* Nichols* stain, is Street Strain 14 (SS14), isolated for the first time in 1977 in Atlanta from the skin lesion of a patient with secondary syphilis.

In 1998* T. pallidum Nichols* strain became one of the first annotated bacterial genomes using Sanger sequencing [6]. In 2008, the genome of the SS14 strain was sequenced by an oligonucleotide array [7]. In 2013 both* T. pallidum Nichols* and SS14 strains were resequenced using next-generation sequencing, and that considerably improved the genome annotation [8].

The genome assembly showed a single circular chromosome of 1,138,006 bp or 1,139,633 bp with a G + C base composition of 52.8% and a total of 1,041 or 1,039 predicted open reading frames (ORFs) in Nichols and SS14 strains, respectively. About 5% of* T. pallidum* genes are specific to the family* Spirochaetaceae*, whereas most of them are genus- and species-specific. Of the 1,041 ORFs in* T. pallidum Nichols* strain, only 577 (55% of total) have predicted biologic functions based on sequence similarities, while 177 ORFs (17%) match hypothetical proteins and 287 ORFs (28%) have no database match and may be novel genes. Among the 1,039 ORFs in genome of* T. pallidum* SS14 strain, functions of 444 genes (43%) were not determined also [9]. Recently, the function of 207 hypothetical proteins was predicted using sequence- and structure-based method and was hypothesized for more 237 genes.

In summary, these results showed one of the smallest bacterial genomes (only few intracellular pathogenic species have a smaller genome) and greatly stimulated the study of this uncultivable* in vitro* bacterium [10].

## 3. *Treponema pallidum* Proteome

The identification of* T. pallidum* proteins began in 1975 with the application of electrophoretic techniques. Based on the SDS-PAGE results the* T. pallidum* protein pattern was described and the nomenclature was firstly standardized [11]. This format consists of the prefix TpN (for* T. pallidum Nichols* strain) followed by a consensus relative molecular mass value. Further, two-dimensional gel electrophoresis (2DGE) technique significantly improved* T. pallidum *proteome research.

In a prominent study of McGill et al. [12] 2DGE was used for* T. pallidum Nichols *strain analyses, which in accordance with amino acid sequence data showed highly expressed proteins. In spite of more than 1000 expected proteins only 148 spots that represented 88 polypeptides were identified by their relative positions in 2DGE patterns, which were high-level expressed genes products.

A recent* T. pallidum* proteome characterization using complementary mass spectrometry technique revealed 557 unique proteins at a high level of confidence, including 106 items firstly accounted at the protein level [13]. These data provide most valuable insights into* in vivo T. pallidum *protein expression representing 54% of the predicted proteome.

The unusual feature of the* T. pallidum* proteome subcellular location was an extremely low density of proteins located in the outer membrane (approximately 1% of the number found in the* E. coli* outer membrane) [14]. OM proteins include the species-specific family of 12* T. pallidum* repeat (Tpr) proteins, and some of them were predicted to be involved in membrane permeability. Other surface-exposed proteins were firstly designated as* Treponema *spp. rare outer membrane proteins (TROMPs), while newly identified OM proteins began to denote Tp prefix followed by ORF number in* T. pallidum *genome (e.g., Tp0326, Tp0453), and this format is now the most common.

Located in periplasmic space flagellar proteins (traditionally denoted as Fla) are widely presented in* T. pallidum* proteome, namely, FlaB1, FlaB2, and FlaB3 proteins of the spiral filament inner core covered with FlaA protein of the outer sheath, complemented with hook-associated and IM located complex of flagellar motor proteins that are typical for* Spirochaetaceae* family [15].

A recent analysis of the* T. pallidum *proteome predicted the presence of a large number of lipoproteins and also a high-level expression of lipoprotein genes [16]. Most of them are located in the inner membrane, where they play a role in nutrient reception and transport or have unpredicted functions.

**Figure 1:**
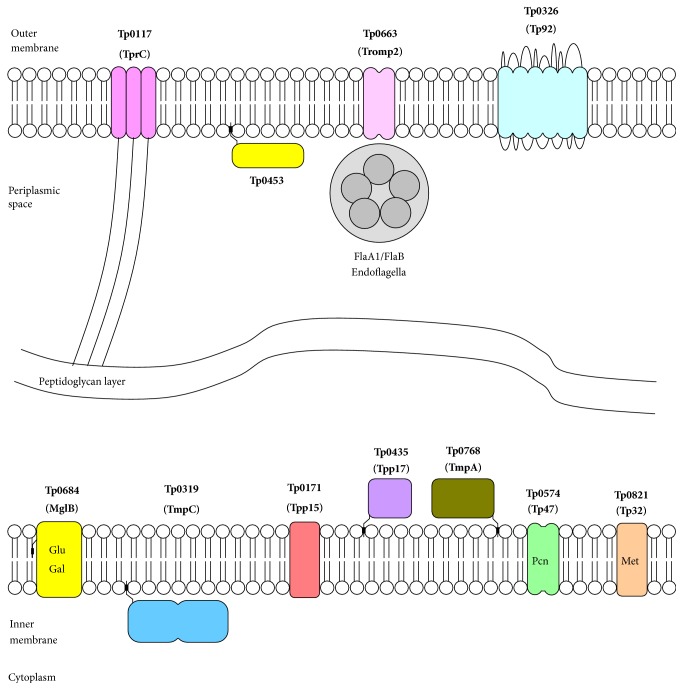
Topological model of* T. pallidum* seroreactive (lipo)proteins proposed localization.

A proposed allocation of* T. pallidum* proteins in treponemal cell membrane is presented in Figure 1.

## 4. *Treponema pallidum* Immunoproteome

**Figure 2:**
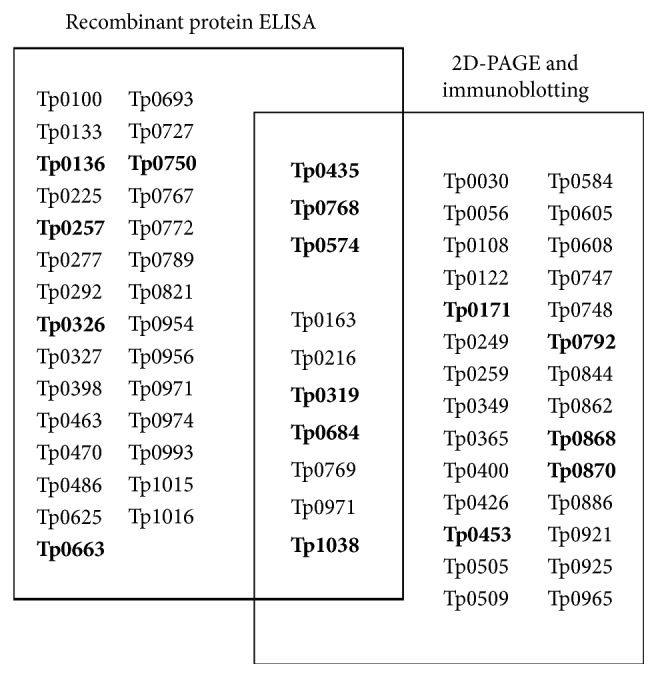
*T. pallidum *proteins, which exhibit immunoreactivity with serum from syphilis patients in Brinkman et al. 2006 (recombinant protein ELISA) and McGill et al. 2010 (2D-PAGE immunoblotting) proteome research. Bold indicates proteins discussed in the present review.

The set of proteins, which induced immune response in the host and showed reactivity with sera from syphilis patients, was termed as* T. pallidum *immunoproteome. In prominent Brinkman et al. [17] and McGill et al. [12] studies that investigated protein expression library and* T. pallidum *strain* Nichols* proteins extracted from testicular tissue of infected rabbits, respectively, only 34–38 reactive antigens were detected. There is no complete identity between these sets of proteins (Figure 2), however principal proteins matched together.

Firstly, some inner membrane lipoproteins were typically reactive with sera from patients at all stages of syphilis, and this subset of seroreactive antigens strictly correlates with Brinkman et al. and McGill et al. immunoproteome studies. Despite the fact that lipoproteins are not exposed on the bacterial surface and are preliminarily located in IM, they are able to induce the high-level immune response, so these were the antigens to use for syphilis diagnostics, and that significantly developed the treponema-specific serological tests.

Secondly, few surface-located antigens were revealed in* T. pallidum* immunoproteome, and that reflects the extremely low density of proteins in the outer membrane of this microorganism. The small number of these potential targets limits the overall detection of the spirochete and allows the pathogen to evade the host immune system, giving its name a “stealth” pathogen [18]. This subset differ in Brinkman et al. and McGill et al. immunoproteome studies, and still OM proteins play a significant role in the outer membrane permeability and adhesion to biopolymers and* T. pallidum* antigenic variability, and that determines current interest to their role in pathogenicity and their diagnostical use.

Taken together, the immunoproteome data suggest the low* T. pallidum* immunogenicity and also allow specifying the most promising antigens for syphilis diagnostics.

## 5. *Treponema pallidum* Recombinant Lipoproteins as a Source for Sensitive and Specific Serological Diagnostics of Syphilis

The initial treponema-specific tests (i.e., assays for detection of specific antibodies) used native or sonically disrupted* T. pallidum* cells as the source of total number of antigens for the fluorescent treponemal antibody-absorbance test,* T. pallidum* particle agglutination, and* T. pallidum* hemagglutination assay [19]; however, this approach was very complicated and expensive due to the inability of* T. pallidum* to be cultured* in vitro*.

Recently the recombinant protein technology promoted considerably the development of treponema-specific laboratory diagnostics [20]. Most typically the* T. pallidum* DNA derived from* Nichols *strain genome is amplified by PCR and inserted into an expression vector and then to* Escherichia coli* cells for expression of fusion proteins with a tag sequence for efficient chromatography purification. Then obtained recombinant proteins were tested as antigens in either enzyme-linked immunosorbent assay (ELISA) or Western blot (WB) format. This approach greatly changed the knowledge of syphilis immunology and together with immunoproteome research led to the selection of optimal antigen combinations for* T. pallidum* serological detection [21].

Several strong immunogenic antigens that induced a high antibody response during syphilis infection and are not cross-reactive with serum from patients with other spirochetal diseases have been identified. In this set the 15 kDa lipoprotein (**Tp15**;* tp0171* gene product) and the major outer membrane 17 kDa lipoprotein (**Tp17**;* tp0435* gene product) are the key members [22]. At the moment the function of Tp15 is still unknown, while Tp17 is characterized as an eight-stranded *β*-barrel protein with a shallow “basin” at one end of the barrel and an *α*-helix stacked on the opposite end [23], which probably plays a role in either protein ligand binding, treponemal membrane architecture maintenance [24], or syphilis pathogenesis by activation of the expression of intercellular adhesion molecule 1 (ICAM-1), E-selectin, and monocyte chemoattractant protein-1 (MCP-1) genes in endothelial cells [25]. Another strong immunogen is 47 kDa lipoprotein (**Tp47**;* tp0574* gene product) which is a carboxypeptidase (major* T. pallidum* penicillin-binding protein) [26] and plays a role in host-pathogen interaction via stimulation of microvessel endothelial cells to synthesize intercellular adhesion molecule and via induction of vascular cell adhesion molecule [27].

The early syphilis diagnostics were based on a single recombinant antigen, where sensitivities and specificities of Tp15, Tp17, and Tp47 were 100% and 96%; 100% and 100%; 100% and 20%, respectively [28], while assays with two or three antigen combinations resulted in the improvement of diagnostic assay [29]. Further an artificial fusion lipoprotein** Tp15-Tp17-Tp47** was established as an instrument for rapid, simple, and convenient syphilis serological screening in the clinical setting using diagnostic ELISA method [30] or miniaturized protein biochip technique [31].

In some other studies the subset of recombinant lipoproteins, which induce the strongest antibody response and are currently used in* T. pallidum *diagnostic tests, includes 44.5 kDa lipoprotein (**TmpA**;* tp0768* gene product) [32] and chimeric* E. coli* expressed Tpp15-Tpp17-Tp44.5-Tp47 antigen (Meridian Life Science, Inc., Memphis, Tennessee USA). More rarely the recombinant products of* tp0319* gene (**TmpC**, 35 kDa purine nucleoside receptor lipoprotein) [33] and* tp0684* gene (**MglB-2**, methylgalactoside ABC transporter, 41 kDa homolog of galactose/glucose-binding lipoprotein) [34] are also used [35, 36] preliminarily in Western blot assay as a combination of these immunogenic antigens [37] and are proved to be highly sensitive and specific for acquired syphilis.

In recent observation the** Tp32** lipoprotein (*tp0821* gene product) was characterized as L-methionine-binding lipoprotein localized in* T. pallidum* inner membrane [38]. Despite the fact that this protein was low reactive in Brinkman et al. immunoproteome study [17] and was not detectably reactive in McGill et al. study [12] the serological tests based on Tp0821 showed 91,0% and 98,3% positive rates of the IgM ELISA and the IgG ELISA, respectively, which correlated with the results of other treponemal tests [38]. The specificity was 94,3–100% when Tp0821 immunoassay was cross-checking with serum samples obtained from 30 patients with Lyme disease, 5 patients with leptospirosis, and 52 uninfected controls. According to these data the authors indicated Tp0821 as a new diagnostic antigen that requires further verification and confirmation.

In summary, treponema-specific tests based on the technology of recombinant lipoproteins significantly improved the diagnostics of syphilis providing an excellent 95–99% sensitivity and specificity of serological assays, being less effective in early and late stages of syphilis diagnostics [21] and in estimating the effect of therapy. Also, they do not distinguish between disease stages, while immunoproteome researches indicate the possibility of differential immune response to certain* T. pallidum *antigens during infection. Thus, the development of enlarged recombinant antigen panel for* T. pallidum *detection remains as actual goal of multidisciplinary molecular biology, microbiology, and immunology research [39].

## 6. Surface-Exposed* Treponema pallidum* Proteins for Improved Serological Diagnostics of Syphilis

The OM proteins are the most important immunological targets due to their availability in intact* T. pallidum* cells; however, they are rare compounds. In Cox et al. study [14] only 17 candidates for OM proteins were identified: 7 members of the Tpr family, 9 non-Tpr hypothetical proteins, and TP0326 (Tp92).

The specific* T. pallidum *repeat (**Tpr**) family of proteins includes 12 members, which can be divided into three subfamilies [40]. Subfamily I includes genes* tprC*,* tprD*,* tprI, *and* tprF*; and subfamily II includes genes* tprE*,* tprG,* and* tprJ *which encode products with common N- and C-termini flanking central domains that differ in sequence and length, while less homologic subfamily III (*tprA*,* tprB*,* tprH*,* tprK*, and* tprL*) differs in variable regions. Subfamily I Tpr proteins [41] possess a conserved sequence at the N- and C-termini and central regions and are predicted to be located in the outer membrane and involved in membrane permeability. For example, the TprC/D (Tp0117/0131) and TprI [42] are proposed to be trimeric, pore-forming proteins with identical *β*-barrels and N-terminal periplasmic domains that directly or indirectly link the barrels to the peptidoglycan layer (Figure 1). An extensively studied** TprK*** (tp0897)* of the III subfamily genes undergoes variation of seven variable regions (V1–V7) by nonreciprocal recombination with a large repertoire of “donor sites” to generate new mosaic proteins [43]. Because the V regions are recognized as the significant targets of the humoral immune response, it can be considered that immune selection of new TprK variants is a mechanism for “antigenic shift” of* T. pallidum *immune evasion and persistence [44].

Surprisingly, despite the strong antibody and T-cell responses against the N-terminal conserved region of the subfamily I Tpr proteins and TprK protein, the Tpr proteins were not indicated as seroreactive in both Brinkman et al. and McGill et al. immunoproteome researches [12, 17]. Probably, this may be determined by the above-described Tpr proteins variability, since TprK sequences differ substantially between and within individual strains, and, as a result, patients often contain multiple* T. pallidum *clones expressing different variants of the* tprK* gene [45]. According to these facts the Tpr family is hypothesized to be essential for* T. pallidum *pathogenesis and evasion from the host immune system, making it a longstanding objective to further vaccine research but limiting its importance as diagnostic antigens.

The group of non-Tpr surface-exposed treponema rare outer membrane proteins, designated as** TROMPs**, includes three members: TROMP-1 (31-kDa), TROMP-2 (28-kDa), and TROMP-3 (65-kDa) proteins. In previous studies [46] TROMPs were shown to be antigenic when tested with serum from infected rabbits and humans; however, in Brinkman et al. immunoproteome research [17]** TROMP-2** (FlaA homolog,* tp0663* gene product) was only reactive with sera from primary-syphilis patients, and in McGill et al. study [12] this protein was identified in* T. pallidum* proteome by 2DGE-MS phoresis but was not found to be reactive with human sera in immunoblot analysis. Recently, recombinant Tp0663 protein was confirmed to be a new serodiagnostic candidate antigen, which was extremely sensitive (98.83%) and specific (100%) for the detection of all stages of the syphilis infection [47].

Another surface-exposed** Tp0326** protein (Tp92;* tp0326* gene product) was described by Cameron et al. [48], using a differential screening strategy to identify* E. coli* clones expressing* T. pallidum* opsonic targets, and later it was characterized as BamA (*β*-barrel assembly machinery protein A) ortholog with the sequence homology to a known Gram-negative family of highly conserved *β*-barrel compounds [49]. Structural modeling of Tp0326 predicted five polypeptide transport-associated (POTRA) domains in the N-terminus and 18-stranded amphipathic *β*-barrel in the C-terminus, which are responsible for the native protein's amphiphilicity [50] (Figure 1). According to Kenedy et al. study related to Tp0326 protein BB0795 of* Borrelia burgdorferi* (Lyme disease spirochete) its function is essential for the assembly of OM proteins [51]. Tp0326 is seroreactive in Brinkman et al. immunoproteome [17], while it did not exhibit reactivity with early latent syphilis sera and did not show immunogenicity in McGill et al. study [12]. This observation can be explained by extremely low level of Tp0326 expression in* T. pallidum* proteome as variation in the antibody responses to POTRA and *β*-barrel portions of this antigen. Surprisingly,* T. pallidum* infected rabbits exhibited an antibody response to both antigenic epitopes, whereas humans with secondary syphilis respond to POTRA only [49]. Recently, Luthra et al. [50] showed that only the *β*-barrel domain of Tp0326 contains surface-exposed epitopes in intact* T. pallidum* and identified an immunodominant large L4 extracellular loop. Based on these results the syphilis diagnostic tests and kits based on Tp0326 recombinant protein, its combination with Tp0453 antigen, and Tp0326-0453 chimeric fusions were developed [52].

**Tp0453** (*tp0453* gene product) is a 287 a.a. protein (putative lipoprotein) associated with the inner surface of* T. pallidum* outer membrane (Figure 1). In Hazlett et al. study [53] this nonlipidated variant of the protein exhibited extensive *β*-sheet structure and amphipathic *α*-helices, whereby when added to artificial bilayers it showed multiple membrane inserting and enhanced the membrane permeability, suggesting being a porin. More recently, the 3D crystal structure of Tp0453 has been solved. It consists of a *α*/*β*/*α*-fold and includes five stably folded amphipathic helices, which are crucial for Tp0453 integration into the membrane [54]. Based on structural dynamics and* Mycobacterium tuberculosis* lipoproteins' comparison data, Tp0453 was proposed to be a carrier of lipids and glycolipids during outer membrane biogenesis. Resuming, Tp0453 is hypothesized to be a novel type of bacterial outer membrane protein, which may render the* T. pallidum* outer membrane permeability to nutrients while remaining inaccessible to antibodies.

Tp0453 is not seroreactive in Brinkman et al. immunoproteome research [17], but in McGill et al. study it showed a moderate seroreactivity and was found to be preliminarily reactive with sera from primary-syphilis patients [12]. This observation correlated with Van Voorhis et al. data [55], which exhibited 100% specificity and sensitivity for Tp0453 in reaction with syphilis patients' sera, giving negative result with relapsing-fever, Lyme disease, or leptospirosis patients' sera. Recently, the methods of producing soluble recombinant Tp0453 via expression in pET28a vector were developed and kits including the soluble and solid substrates, containing Tp0453 protein, are also provided. The development of ELISA method using Tp0453 recombinant products and chimeric Tp0453-Tp0326 proteins as diagnostic antigens are reported. The sensitivities of Tp0453 and the Tp0453-Tp0326 chimera were found to be 98% and 98%, respectively, and the specificities were 100% and 99%, respectively, which characterized these proteins as novel candidate antigens for treponema-specific serological diagnostics [52]. Now Tp0453 together with conventional Tp15, Tp17, Tp47, TmpA, and novel Tp0257 (Gpd) antigens is included into the panel of commercially available WB kit Recom Blot Treponema IgG/IgM 2.0 (Mikrogen GmbH, Germany).

## 7. Novel* Treponema pallidum* Derived Recombinant Products: Adhesins and Periplasmic and Flagellar Proteins—Opportunities and Limitations

**Tp0155** and** Tp0483** were predicted as two putative adhesins of the* T. pallidum *genome and then demonstrated specific attachment to fibronectin and blockage of a bacterial adherence to fibronectin-coated slides [56]. Interestingly, Tp0155 preferentially binds to the matrix form of fibronectin, whereas Tp0483 binds to both the soluble and matrix forms, which exist in different conformational forms with cryptic epitopes becoming exposed during fibronectin matrix assembly. Recently Tp0155 was described as a protein comprising a leader peptide, two N-terminal LysM domains, which recognize carbohydrate polymers, and M23 peptidase sequence, which makes this protein able to degrade peptidoglycan and exhibit the enzymatic activity [57]. In turn, the analyses of Tp0483 outer membrane protein revealed two fibronectin binding regions between 274–289 and 316–333 amino acids residues [58]. In addition to the adhesive and enzymatic functions both* T. pallidum* proteins induce production of IL-6, IL-1*β*, and TNF-*α* in macrophages, and that is associated with the activation of NF-*κ*B [59].

Surprisingly, these proteins were not seroreactive in immunoproteome researches, and in comparative study with Gpd both Tp0155 and Tp0483 [55] gave positive result with only 9% of syphilis patient sera, and all of these reactive sera were from the individuals with early primary infection.

**Tp0136** is 485 a.a., 49 kDa hypothetical protein/lipoprotein exposed on the* T. pallidum* outer membrane. The recombinant protein study revealed the ability to bind fibronectin and laminin glycoproteins, which involved Tp0136 attached to the host extracellular matrix components [60]. Recently it was shown that Tp0136 adheres more efficiently to cellular than to plasma fibronectin via its N-terminal conserved region [61]. Additionally, Tp0136 is highly transcribed during an experimental infection in parallel with the host immune response to the pathogen, which suggests a possible role for this protein in* T. pallidum* persistence.

TP0136 is not reactive in McGill et al. proteome; however in Brinkman et al. study this protein exhibits reactivity to human sera compared to rabbit sera [17] preliminarily with primary-syphilis stage. Recently, the Tp0136 selective fragment (Tp0136B) with a molecular weight of about 28 kDa was tested with sera from primary-syphilis patients, and the positive result was shown in 85.5% of cases [62].

**Tp0751** firstly was described as 237 a.a., 25,8 kDa protein and then was identified as* T. pallidum* laminin-binding adhesin [63], which is crucial for pathogen dissemination in the host organism due to the attachment to the extracellular matrix component laminin—major glycoprotein found within mammalian basement membrane. The laminin-binding region in Tp0751 is limited to 10-amino acid fragment, and this motif inhibited the attachment of* T. pallidum* to laminin, as well as Tp0751-specific antibodies inhibit the attachment of* T. pallidum* to laminin too. Further studies showed the Tp0751 bifunctionality including fibrin clot degradation capability and characterized this molecule as the treponemal metalloprotease pallilysin [64]. Cotranscribed protein** Tp0750** was described as a serine protease, which degrades major clot components (fibrinogen and fibronectin) [65], and was hypothesized to work in cooperation with Tp0751 and together to play a role in* T. pallidum* invasion and dissemination in the host organism.

Despite the significance for* T. pallidum* pathogenicity, Tp0751 is not seroreactive in both abovementioned immunoproteome researches, while Tp0750 exhibited a week seroreactivity in Brinkman et al. study with sera from primary and early latent syphilis [17]. Finally, in comparative studies Tp0751 revealed lower seroreactivity than Tp0257 (Gpd) and Tp1038 (TpF1) [55]. However, being limited for diagnostic use, the Tp0751 protein showed good immunoprotective properties, allowing it to be considered as a promising syphilis vaccine candidate [66].

Firstly** Tp0257** protein was identified as a potential immunoreactive antigen using a differential immunologic expression library screening. According to the results of nucleotide sequence analysis this protein was demonstrated to be the 356-residue homologue of glycerophosphodiester phosphodiesterase (Gpd) [67], an enzyme, that hydrolyzes deacylated phospholipids to alcohol and glycerol-3-phosphate, previously identified in* Haemophilus influenzae*,* Escherichia coli*,* Bacillus subtilis* and* Borrelia hermsii*. The characterization of the recombinant protein Tp0257 showed its bifunctionality, revealing both the enzymatic activity and the capability of binding the Fc-fragment of human IgA, IgD, and IgG immunoglobulins [68]. Initially Tp0257 was predicted to be lipid-modified, associated with the outer membrane and surface exposed, and thus this protein was supposed to play a role in enabling the* T. pallidum *to evade the immune response limiting the antibodies' cytotoxic and opsonic capacities (like its homolog in* H. influenzae*). However, in further analysis it turned to have a subsurface localization (like its homolog in* E. coli*), where substantial portion of this periplasmic polypeptide is associated with peptidoglycan layer.

In Brinkman et al. immunoproteome [17] Tp0257 was reactive with sera from primary, secondary, and early latent syphilis patients but was not detected by 2DGE immunoblotting method in McGill et al. study [12]. There are few data about the diagnostic capacities of recombinant Tp0257 in syphilis serology as an included antigen together with Tp0453 [55].

**Tp1038** (TpF1, antigen 4D, antigen C1–5) is homodecamer comprising 12 identical 19-kDa subunits linked by disulfide bonds, which form a nearly spherical shell [69]. This protein plays a role in iron uptake and functionally belongs to bacterioferritins' group. Moreover, Tp1038 plays a pivotal role in driving an immune response by activation of inflammasome, promoting the development of regulatory T-cells, modulating the release of specific cytokines by monocytes, and stimulating an angiogenesis that is typically observed during secondary syphilis [70].

Tp1038 is seroreactive in Brinkman et al. immunoproteome [17] study with sera from early latent syphilis, whereas in McGill et al. study [12] oligomeric form of this antigen exhibited high antibody responses with all stages of syphilis while it did not exhibit serologic reactivity against the monomeric form. Currently this antigen has shown a high sensitivity (93.3–100%) for the detection of all stages of syphilis and was extremely specific (100%) when tested against potentially cross-reactive sera, and that proposes Tp1038 to be a promising candidate for the screening of syphilis [71].

Flagellar proteins form a significant part of* T. pallidum* proteome being represented by** Tp0868** (FlaB1, 34.5 kDa),** Tp0792** (FlaB2, 33 kDa), and** Tp0870** (FlaB3, 31 kDa) filament core proteins covered with** Tp0249 **(FlaA1) sheath protein, also complemented with hook-basal body complex proteins (**Tp0398** and** Tp0727**) and flagellar motor proteins (**Tp0400**) [72]. Now it is hypothesized that Tp0249 (FlaA1) protein is in a contact with Tp0663 (FlaA2), localized in the inner membrane, and designated as Tromp2 also. These proteins provide a characteristic corkscrew motility, which is significant for* T. pallidum* invasion and dissemination in the host organism.

The hook-basal body complex proteins Tp0398 and Tp0727 are seroreactive in Brinkman et al. immunoproteome [17], while in McGill et al. study [12] a number of proteins, namely, Tp0249, Tp0868, Tp0792, and Tp0870 (FlaA, FlaB1, FlaB2, and FlaB3, resp.), and flagellar motor protein Tp0400 (FliG), were highly seroreactive at all stages of syphilis. In early researches some cross-reactions of* T. pallidum* flagellar proteins with a number of proteins of distantly related spirochaetes were observed [73], and that restricted their diagnostic significance. Recently, a screening of recombinant flagellar proteins showed that FlaB1, FlaB2, and FlaB3 revealed higher overall sensitivity and specificity for IgG antibody with 95.4% and 98.9%; 92.6% and 95.8%; 95.1% and 95.8%, respectively [74]. In addition, FlaB1, FlaB2, and FlaB3 proteins demonstrated an excellent performance for detecting IgM antibody in primary and congenital syphilis, with sensitivity and specificity of 76.8% and 83.1%; 72.0% and 87.7%; 74.4% and 89.2%, respectively. These results put FlaB1, FlaB2, and FlaB3 proteins into the group of novel candidate antigens for syphilis serodiagnostics.

Thus, current studies (summarized in Table 1) show both opportunities and limitations of novel recombinant antigens of* T. pallidum* for the serological diagnostics of syphilis. Most typically some of these antigens (Tp0136, Tp0257, and Tp1038) are more useful for primary and early stages syphilis detection, while overall they are less sensitive (Tp0155, Tp0483, and Tp0751) or less specific (Tp0868, Tp0792, and Tp0870) than conventional immunodominant* T. pallidum *lipoproteins.

## 8. Future Directions in* Treponema pallidum* Recombinant Proteins Development

The recombinant protein technique can provide a significant quantity of highly purified* T. pallidum* antigens for diagnostic use. This led to great progress in a treponema-specific serological tests reliability based on the detection of antibodies against* T. pallidum* immunodominant lipoproteins Tp15, Tp17, Tp47, and some others. The modern “traditional” and “reverse” algorithm use this approach as the second- or the first-line tests, which are effective in most cases of syphilis diagnostics.

Remaining difficulties in syphilis serological screening are related to early forms (without expressed immune response) or late forms of this disease (when immune response decreases as a result of* T. pallidum* migration in the “immunologically privileged” niches). This situation makes it necessary to recruit new additional treponemal recombinant antigens that will be seroreactive in cases of ineffectiveness of immunodominant lipoproteins. For example, development of surface-exposed proteins Tp0326 (Tp92) and Tp0453 have increased sensitivity and specificity of serological tests to 98–100%, especially at primary syphilis stage.

Current research studies revealed numerous novel recombinant antigens, which are promising candidates for the improved syphilis serological diagnostics. However, ongoing studies showed the depletion of diagnostic antigens resource, where some novel products are less effective than conventional recombinant lipoproteins. The period of rapid progress in syphilis serodiagnostics development ended and each new success in this field requires a lot of experimental and clinical efforts.

On the other hand, the current syphilis laboratory diagnostics paradigm, which provides alternative diagnostic result (“yes” or “no”), significantly reduces the experimental research area, while the immunoproteome studies indicate greater possibilities for serological analyses compared to screening and confirmatory tests only. Changes in behavioral strategy of* T. pallidum* in the course of the disease determined by its interaction with the immune system and manifested in different proteins expression profiles may be a clue for the differentiation of syphilis stages based on detection of antibodies against these variable antigens. For example, some novel* T. pallidum* recombinant antigens (like Tp0136, Tp0155, Tp0483, and others) that are not reactive in all syphilis stages and in current paradigm are assessed as less effective for syphilis screening are promising candidates for new generation of immunotests with advanced diagnostic capabilities. In line with these expectations the quantitative and qualitative evaluation of antibody level against certain antigens may reveal a variable immune response (fingerprints) that is typical for different syphilis stages.

Another unresolved issue is the continued reactivity of modern conventional treponemal tests for a long time after the syphilis cure that determines their inefficiency for monitoring of the treatment response, relapse, or reinfection in previously treated patients. Currently this goal is achieved using the low-specific nontreponemal tests, which contrasts with the experience of the serological monitoring of other infectious diseases. In this context, the search for novel* T. pallidum* antigens, whose antibodies are rapidly eliminated from host blood flow after the pathogen's eradication, is another promising direction in recombinant proteins' development.

Thus, the development of enlarged panel of* T. pallidum* recombinant antigens remains an actual task of multidisciplinary biomedical research. The high-resolution methods (immunoblot or immunochip) and multicentral examination protocols allow determining the number of antigens in the panel with the following criteria: (i) detectable immunoreactivity; (ii) expression level on different syphilis stages; (iii) decreasing immune response after the infection regress. Scan of these recombinant antigens and their application for new aspects of syphilis serological diagnostics are the tasks of the future research studies.

## Figures and Tables

**Table 1:** *T. pallidum* proteins used and proposed for syphilis serological diagnostics.

Gene (ORF number)	Protein name	Protein description	Immunoproteomic data		Seroreactivity at syphilis stages	Sensitivity/specificity; (% of positive result)	Links
			Brinkman et al. [17]	McGill et al. [12]			
*Inner membrane lipoproteins*							
*tp0171*	Tp15	15 kDa lipoprotein	None	+++	All stages	100/100	[28]
*tp0435*	Tp17	17 kDa lipoprotein	9,6–16,6	+++	All stages	96/100	[28]
*tp0574*	Tp47	47 kDa penicillin- binding protein, carboxypeptidase	2,9–10,0	+++	All stages	100/20	[28]
*tp0768*	TmpA	44.5 kDa lipoprotein	8,2–15,3	+++	All stages	76–100/ 99,6	[32]
*tp0319*	TmpC	35 kDa lipoprotein, purine nucleoside receptor A lipoprotein	2,8–6,2	+/++	All stages	100/100	[34]
*tp0684*	Tp38, MglB-2	38 kDa lipoprotein, methylgalactoside ABC transporter, galactose/glucose-binding lipoprotein	6,8–19,0	+++	All stages	ND	[35]
*tp0821*	Tp32	32 kDa lipoprotein, L-methionine-binding lipoprotein	1,0–1,7	None	All stages	91,0–98,3/ 94,3–100	[38]

*Surface-exposed and outer membrane associated proteins*							
*tp0897*	TprK	Heterogenic antigen variable by gene conversion	None	None	ND	ND	[43] [44] [45]
*tp0663*	TROMP-2	28-kDa outer membrane protein, FlaA homolog	0,8–1,9	None	All stages	98,83/100	[47]
*tp0326*	Tp92	BamA (*β*-barrel assembly machinery protein A) ortholog	1,2–2,6	None	Mostly at primary stage; lower reactivity in secondary and early latent stage	86/99 98/97	[52] [55]
*tp0453*	Tp0453	Proposed carrier of lipids and glycolipids	None	+/++		98/100 100/100	[52] [55]

*Adhesins*							
*tp0155*	Tp0155	Binds to the matrix form of fibronectin and exhibit peptidase enzymatic activity	None	None	low reactive at primary stage	ND (27,9% positive)	[55]
*tp0483*	Tp0483	Binds to both the soluble and matrix forms of fibronectin	None	None	Low reactive at primary stage	ND (41,8% positive)	[55]
*tp0136*	Tp0136	49 kDa outer membrane (lipo)protein; it binds to fibronectin and laminin	0,7–2,1	None	Primary stage	ND (85,5% positive)	[62]
*tp0751*	Tp0751	25,8 kDa protein; it binds to laminin and exhibits metalloprotease activity	None	None	ND	ND (41,8% positive)	[55]
*tp0750*	Tp0750	Cotranscribed with Tp0751 serine protease	0,8–2,1	None	Primary and early latent stages	ND	[65]

*Putative periplasmic proteins*							
*tp0257*	Gpd	Glycerophosphodiester phosphodiesterase, binds Fc-fragment of human IgA, IgD, and IgG immunoglobulins	3,0–7,3	None	All stages	91/93	[55]
*tp1038*	TpF1, 4D, C1–5	bacterioferritin, homodecamer from 19-kDa subunits	0,8–2,2	+++	All stages	93–100/ 100	[71]

*Flagellar proteins*							
*tp0868*	FlaB1	Flagellar filament 34.5-kDa core protein	None	+++	All stages	95.4/98.9	[73]
*tp0792*	FlaB2	Flagellar filament 33-kDa core protein	None	+++	All stages	92.6/95.8	[73]
*tp0870*	FlaB3	Flagellar filament 31-kDa core protein	None	+++	All stages	95.1/95.8	[73]
